# Link between Cancer and Alzheimer Disease via Oxidative Stress Induced by Nitric Oxide-Dependent Mitochondrial DNA Overproliferation and Deletion

**DOI:** 10.1155/2013/962984

**Published:** 2013-04-03

**Authors:** Gjumrakch Aliev, Mark E. Obrenovich, Shams Tabrez, Nasimudeen R. Jabir, V. Prakash Reddy, Yi Li, Geoffrey Burnstock, Ramon Cacabelos, Mohammad Amjad Kamal

**Affiliations:** ^1^GALLY International Biomedical Research Consulting LLC, 7733 Louis Pasteur Drive, No. 328, San Antonio, TX 78229, USA; ^2^School of Health Science and Healthcare Administration, The University of Atlanta, 6685 Peachtree Industrial Boulevard, Atlanta, GA 30360, USA; ^3^Departments of Chemistry and Biology, Cleveland State University, 10701 East Boulevard, 113-W, Cleveland, OH 44106, USA; ^4^Metabolomics and Enzymology Unit, Fundamental and Applied Biology Group, King Fahd Medical Research Center, King Abdulaziz University, Jeddah 21589, Saudi Arabia; ^5^Department of Chemistry, Missouri University of Science and Technology, 341 Schrenk Hall, Rolla, MO 65409, USA; ^6^Department of Genetics, School of Medicine, Yale University, 333 Cedar Street, New Haven, CT 06520, USA; ^7^Autonomic Neuroscience Institute, Royal Free Hospital School of Medicine, London NW3 2PF, UK; ^8^The Department of Pharmacology, Level 8, Medical Building (No. 181), Corner of Grattan Street and Royal Parade University of Melbourne, Victoria, 3010, Australia; ^9^EuroEspes Biomedical Research Center, Institute for CNS Disorders and Genomic Medicine and Camilo José Cela University, Sta. Marta de Babío, s/n, La Coruña, 15165 Bergondo, Spain

## Abstract

Nitric oxide- (NO-) dependent oxidative stress results in mitochondrial ultrastructural alterations and DNA damage in cases of Alzheimer disease (AD). However, little is known about these pathways in human cancers, especially during the development as well as the progression of primary brain tumors and metastatic colorectal cancer. One of the key features of tumors is the deficiency in tissue energy that accompanies mitochondrial lesions and formation of the hypoxic smaller sized mitochondria with ultrastructural abnormalities. We speculate that mitochondrial involvement may play a significant role in the etiopathogenesis of cancer. Recent studies also demonstrate a potential link between AD and cancer, and anticancer drugs are being explored for the inhibition of AD-like pathology in transgenic mice. Severity of the cancer growth, metastasis, and brain pathology in AD (in animal models that mimic human AD) correlate with the degree of mitochondrial ultrastructural abnormalities. Recent advances in the cell-cycle reentry of the terminally differentiated neuronal cells indicate that NO-dependent mitochondrial abnormal activities and mitotic cell division are not the only important pathogenic factors in pathogenesis of cancer and AD, but open a new window for the development of novel treatment strategies for these devastating diseases.

## 1. Introduction

Mitochondrial decay has been postulated to be a significant feature underlying aging and age-related disease processes [1]. Mitochondrial dysfunction and free radical-induced damage play a significant role in the pathogenesis of tumors, tumor-growth, metastasis, and cellular and tissue aging [2]. Decline in mitochondrial function most likely leads to cellular energy deficits, especially during situations known to require increased energy demand and in organs or tissues where the energy needs and metabolic demand are particularly high, such as in the brain or fast-growing tumors. These deficits can compromise vital adenosine triphosphate- (ATP-) dependent cellular functions, such as detoxification, system repair, DNA replication, ATP-dependent protein degradation, and osmotic balance. As a result of this increased energy demand coupled with hypoxia and oxidative stress, some tumors switch to glycolysis to meet energy demands. Similarly, defective ATP production and increased generation of reactive oxygen and nitrogen species (ROS and RNS) may induce mitochondrial-dependent cell death as the damaged mitochondria are unable to maintain the energy demands of the cells [1].

## 2. Physiological Roles of NO and NO Synthase (NOS)

NO, a free radical species, is a well-known physiological signaling agent, and a pleiotropic regulator in various pathologies including tumor growth and AD [2, 3]. It is synthesized by nitric oxide synthase (NOS) enzymes by transforming L-arginine to L-citrulline. NOS enzymes comprise inducible NOS (iNOS or NOS2), endothelial NOS (eNOS or NOS3), and neuronal NOS (nNOS or NOS1) [2–6]. Various studies have shown that each of the three isoforms may be implicated in either promotion or inhibition of human cancer development. High amounts of iNOS expression, caused by activated macrophages, may be cytostatic or cytotoxic for tumor cells; in contrast, low activity may have an opposite effect and promote tumor growth [2, 6]. In fact, nitric oxide may play a crucial role in mitochondrial respiration [4–6], since even low (nanomolar) concentrations of NO were found to reversibly inhibit the mitochondrial respiratory chain enzyme cytochrome oxidase (complex IV) and compete with molecular oxygen. Inhibition of cytochrome oxidase by NO results in the reduction of the electron-transport chain, and favors the formation of the superoxide radical anions (O_2_^−^). NO upon reaction with superoxide radical anion forms peroxynitrite (ONOO^−^), which is more cytotoxic than NO itself [2, 3, 7]. Peroxynitrite has been identified as a potent oxidant and potential mediator of vascular tissue injury [3] and cell death [3, 7]. Several laboratories have investigated the cellular consequences of endogenously generated and exogenously applied NO [4–6]. Accumulating evidence demonstrates that endogenous NO (using endothelial cells (EC)), basally produced or generated in response to stimulation with bradykinin, reduces the rate of oxygen consumption by the cells [8]. This finding suggests that endogenous NO modulates oxygen consumption under basal and stimulated conditions and leads to the formation of reactive oxygen species, O_2_^∙−^ [6]. Moreover, an extended study by the Moncada's group has found that prolonged exposure to exogenous NO results in persistent inhibition of mitochondrial respiration, which is localized mainly at complex I [4–6]. This persistent inhibition seems to be the result of oxidative stress generated from mitochondrial free-radical generation and involves S-nitrosylation of mitochondrial complex I. Indeed, inhibition of the respiratory chain causes its reduction and the subsequent generation of superoxide anions (vide supra). It is likely that these anions are initially converted by superoxide dismutase to hydrogen peroxide, which is known to be a transcription factor of several defense genes. If this inhibition is prolonged, it may result in the generation of peroxynitrite at the site of superoxide anion production [6]. Thus, persistent inhibition of cytochrome oxidase could elicit a two-stage response, an early one in which the main consequence is the release of small amounts of hydrogen peroxide (H_2_O_2_), and a later one that involves higher concentrations of H_2_O_2_ and formation of peroxynitrite. However, the mitochondrial DNA overproliferation under these conditions is unknown [3]. Nevertheless, many of the biological effects attributed to NO can be mediated by peroxynitrite [3]. Moreover, superoxide and NO can be produced simultaneously in close proximity, which leads to increased peroxynitrite formation [7]. Even modest increases in both superoxide and NO formation at a 10-fold greater rate increase peroxynitrite formation by 100-fold. Under proinflammatory conditions, simultaneous production of superoxide and NO is rapidly activated thereby increasing the production rates by 1,000-fold, which consequently increase the formation of peroxynitrite by up to 1,000,000-fold. The role of NO-induced mitochondrial failure in the pathogenesis of tumors, particularly tumor angiogenesis, now has been widely accepted. Recently, an aging rat model of chronic brain hypoperfusion (CBH) that mimics human mild cognitive impairment (MCI) was used to examine the role of NOS isoforms on spatial memory function. In one of our studies, rats with CBH underwent bilateral common carotid artery occlusion (2-vessel occlusion (2-VO) were compared with nonoccluded sham controls (S-VO)) [9]. After the administration of neuronal and endothelial (nNOS/eNOS) constitutive inhibitor nitro-L-arginine methyl ester (L-NAME) only 2-VO rats worsened the ability of their spatial memory [9]. Our findings indicate that vascular NO derived from eNOS plays a critical role in spatial memory function during CBH, possibly by keeping cerebral perfusion optimal through its regulation of microvessel tone and cerebral blood flow. This study could lead to the identification of therapeutic targets for preventing MCI and treatment of AD [9].

The role for NO-dependent process is quite clear in AD pathogenesis and remodeling of cortical cholinergic system through degradation of mature nerve growth factor (NGF) in AD. It is also well established that the cortical cholinergic system plays a crucial role in cognitive processing and memory formation [10, 11]. Pharmacological evidence of cholinergic atrophy and metastasis depends on matrix metalloproteinases in both diseases. In an activity-dependent manner NGF precursor forms proNGF, along with the convertases and proteases necessary to form mature NGF (mNGF) and to degrade the free, unbound mNGF by serine protease involved the matrix metalloproteinase 9 (MMP-9) [12]. However, the exact cellular mechanisms behind tumor vascular growth and the relationship to NO oxidation products, such as nitrotyrosine products, lipid peroxidation, as well as mitochondrial DNA (mtDNA) deletion remains unknown [3].

## 3. The Role of Endothelin Signaling

During conditions favoring hypoxia, hypoxia-induced transcription factor (HIF-1) binds to the hypoxia response element (HRE) in the endothelin-1 (ET-1) promoter region to induce ET-1 transcription [13]. In response to hypoxia, oxidized low-density lipoprotein (LDL), cytokines, and ET-1 levels are upregulated in EC [14]. The most extensively studied member of the endothelin system or the so-called endothelin axis and its expression is induced by various cytokines and stimuli [14, 15], such as TNF stimulated ET-1 secretion in cultured bovine airway smooth-muscle cells (SMC) and human airway epithelial cells [16, 17]. The TNF superfamily of cytokines are particularly important in cancer progression and apoptosis.

The endothelin axis includes three endothelins (ET-1, ET-2, and ET-3), which are widely expressed in various human tissues including brain, skeleton muscle, testis, pancreas and have similar structure [14] (along with two G-protein-coupled endothelin receptors (ET-RA and ET-RB), two proteinases) as endothelin converting enzymes (ECE-1 and ECE-2) [18]. ET-1 and ET-2 bind to ET-RA more avidly than ET-3, while all three endothelins have similar affinity for ET-RB. When the primary physiological function of endothelins was tested in arteries and veins, ET-1 and ET-2 induce equal maximum contraction and potent responses, whereas ET-3 induces lower maximum contractions and overall a less potent response [19]. Likewise, ET-3 is a factor that attenuates ET-1 signaling through ET-RA. ET-RA is found predominantly in smooth and cardiac muscle cells, whereas ET-RB is highly expressed in EC. ET-RA primarily mediates vasoconstriction, contraction, and proliferation induced by ET-1 [20] and is found predominantly in EC, where it mediates endothelium-dependent vasodilation through NO and prostacyclin. ET-RB is believed to have multiple effects, including EC survival, NO production, and ET-1 clearance [20]. Both ET-RA and ET-RB mediate antiapoptotic effects in human SMC [21].

In many cases, increased levels of ET-1 and the receptors (ET-RA, ET-RB) are detected in tumor tissues [14]. It has been reported that ET-RA mediates ET-1 induced cancer cell proliferation [22] and promotes epithelial-to-mesenchymal transition [23]. ET-RB may mediate antiapoptosis effect induced by ET-1 [24]. The loss of ET-3 expression due to epigenetic inactivation has been reported in human breast cancer by measuring mRNA levels [25]. This is consistent with the speculation that ET-3 may attenuate ET-1 signaling through ET-RA. It favors ET-1 signaling through ET-RA when ET-3 is decreased in cancer cells. In order to understand the detailed functions of endothelins in cancer, signaling pathways in tumor cells initiated by the two different receptors need to be further explored. Diverse signaling pathways from the two different receptors can be the reason why endothelins and the receptors are regulated differently in tumor tissues.

Endothelin signaling is speculated to be involved in cell differentiation, proliferation, migration, and angiogenesis in tumors [25]. It has been demonstrated that ET-1 is overexpressed in various tumor tissues [22], including prostate tumor and high grade prostatic intraepithelial neoplasia [26], breast cancer [27], and lung tumor [28]. Expression of mRNA of ET-1, ET-RA, and ET-RB was detected in ovarian carcinoma cell lines HEY and OVCA 433 by RT-PCR, and secreted ET-1 was detected in the culture media by ELISA [22]. ET-1 and ET-RA are overexpressed in canine ovary tumors [29], which is consistent with the function of ET-RA signaling induced by ET-1 that is involved in cell proliferation. However, transfection often results in a supraphysiological level of expression of target genes, which may induce some artifacts such as increased formation of heterogeneous dimers between ETA and ETB receptors [30].

Although antagonists of endothelin receptors for the treatment of cancer are not in clinical development, specific peptide-based antagonists of ET-RA and ET-RB have been used in *in vitro *and *in vivo* cancer studies. Cancer cell proliferation was reported to be inhibited when ET-RA was specifically blocked in colorectal cancer cell lines [31]. When orally active high affinity ET-RA antagonist ZD4054, which has no detectable affinity for ET-RB, was applied *in vitro*, it inhibited ET-1 induced proliferation of human pre-osteoblast cells [21], human ovarian carcinoma cell lines HEY and OVCA 433 [22] and further demonstrated ET-RA was involved in signaling in cancer cell proliferation.

ET-1 is a potent vasoconstrictor that induces contraction at nanomolar concentrations in several vascular beds. Aliev and coworkers and others have identified multiple inducers of cell cycle reentry, ectopic cell cycle marker and ET-1 overexpression, as a hallmark of cancer, which is also involved in AD [2, 3, 32]. The complex neurodegeneration mechanism underlying AD, although incompletely understood, is characterized by an aberrant neuronal cell cycle reentry. While cell cycle is not the focus of the present paper, the oncogenic parallel between AD and cancer, especially in the context of vascular content, is one the focus of this communication. The pathological evidence of ectopic cell cycle marker and cell cycle regulatory proteins expression in AD suggests that cell cycle reentry is an earlier event, which occurs at prodromal stages, that is, those stages which show formation of either amyloid-beta (A*β*) plaques or neurofibrillary tangles (NFTs) as a hallmark for human AD and/or AD-like pathology in transgenic mice [3, 32]. In this regard, Aliev and coworkers have already demonstrated mitochondrial DNA deletion as well as mitochondrial structural abnormalities in the vascular walls of the human AD, yeast artificial chromosome (YAC), and C67B6/SJL transgenic positive (Tg+) mice overexpressing amyloid amyloid-*β* precursor protein (A*β*PP) [33]. We expect similar findings in case of cancer as well, which most likely could have an even more important role in cytotoxicity and hypoxia adaptation by primary and metastatic cancerous cells, as well as within the aging tissues including brain itself. In this regard, recent advances in understanding the pathogenesis of cell cycle reentry which relates the pathogenesis in AD as well as cancer deserves special attention. Key in the AD pathogenesis is NO formation and release from vascular and immunologic cells and its conversion to peroxynitrite, which nitrates tyrosine residues of enzymes, and causes mitochondrial DNA damage [2, 3, 32]. Moreover, Aliev and coworkers' findings present a strong case for the role of NOSs, ET-1, and their oxidation products such as nitrotyrosine activity in the development of human colorectal cancer metastasis to liver and in malignant brain cancers [2].

## 4. GRK2 is Upstream in Endothelin Signaling Cascades

**Figure 1:**
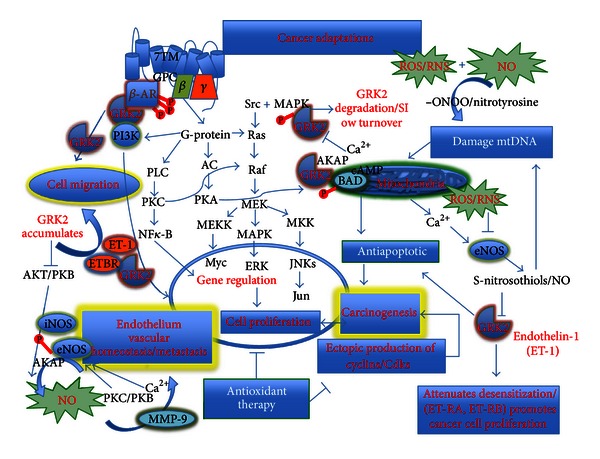
The hypothetical schematic drawing the potential role of GRK, MAPK, JNK, and p38 in the adaptive response during the development and metastasis of the tumorigenesis.

NO production by EC seems to be regulated via Akt/PKB signal transduction pathway, which activates eNOS. Akt physically interacts with GRK2 and inhibits Akt activity and its phosphorylation and thus production of NO [34]. In the aforementioned study, GRK2 expression increased in sinusoidal endothelial cells from portal hypertensive rats and knockdown of GRK2 restored Akt phosphorylation and NO production, and normalized portal pressure. Thus, an important mechanism underlying impaired activity of eNOS in injured sinusoidal EC was found to be defective phosphorylation of Akt caused by overexpression of GRK2 after injury (Figure 1).

**Figure 2:**
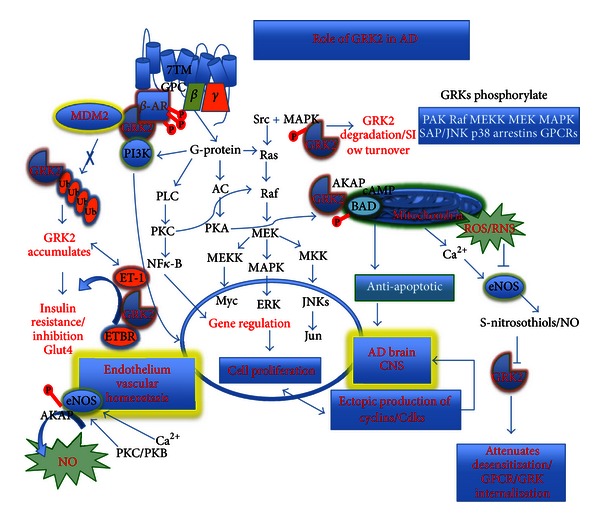
The schematic drawing pattern of the GRK2 overexpression that most likely appeared to be as a compensatory to the hypoxia and hypoperfusion induced oxidative stress that initiates the development and maturation of AD. Modified and reprinted with permission of CNS Neurol Disord Drug Targets [92].

Some of us and others [35–38] have also found a critical role of GRK2 in the endothelin signaling cascade and many of the effects of ET-1 on cancer may be mediated by GRK2 (Figure 2). In this regard, it is important to note the importance of GRK2 on ET-1 receptors, downstream events, and its relationship with cancer. The importance of the strong immunologic relationship to most cancers is illustrated in part by high expression of GRK2 in different cellular types of the immune system. This emerges as an important regulator of cell responses during inflammation, such as leukocyte trafficking to the inflammatory foci, T-cell egression from lymphoid organs, leukocyte activation, or proliferation [39]. GRK2 is known to phosphorylate chemokines and chemotactic receptors for CCR5, CCR2b, CXCR4, CXCR2, and substance P, S1P or formyl-peptide, respectively [39].

Aberrant epithelial cell motility plays a key role in cancer progression and metastasis. GRK2 expression levels might alter migratory responses in pathological conditions (Figures 1 and 2). A potential role for GRK2 in epithelial cell migration was investigated by Penela and colleagues, where GRK2 was found to promote actin cytoskeletal changes and paxillin localization consistent with enhanced focal adhesion turnover and higher cell motility [40]. These authors further found that GRK2 promotes increased migration towards fibronectin in different epithelial cell lines and in fibroblasts, where these effects were independent of GRK2 kinase activity. The contrary has been described in immune cells, where increased GRK2 expression facilitates migration towards fibronectin and GRK2 downregulation impairs migration of the epithelial cells [40]. GRKs seem to be a new target for therapeutic intervention. In addition to a currently available ET receptor antagonist, overexpression of GRK2 attenuated ET-induced SMC proliferation and ETA receptor desensitization mechanisms in vascular SMCs [35]. Guo and colleagues [41] showed TGF*β*-induced GRK2 expression attenuates Angiotensin II-regulated vascular smooth muscle cell proliferation and migration. GRK2 acts through a negative feed-back loop mechanism to terminate TGF-induced SMAD signaling. Activation of the TGF*β* signaling cascade in VSMCs results in increased GRK2 levels and inhibits Angiotensin II-induced ERK phosphorylation, and antagonizes Angiotensin II-induced VSMC proliferation and migration at Mek-Erk interface [42]. Although ET-1 can elicit prolonged physiologic responses, GRKs most likely initiate ET-R desensitization. Moreover, endothelin A and B receptors (ETA-R and ETB-R, resp.) can be regulated indistinguishably by GRK-initiated desensitization. Furthermore, GRK2 and platelet-derived growth factor (PDGF) was reported to attenuate SMC proliferation [43].

Finally, emerging evidence points a role of GRK2 as both an extrinsic and intrinsic cell-cycle regulator. GRK2 expression is reported to have distinct impact on cell proliferation and mitogenic signaling depending on both the cell type and the mitogenic stimuli (Figures 1 and 2). It also has diverse regulatory roles directly related to cancer. The complex functional interaction networks during cell cycle progression that are critical at particular stages of the cell cycle and in cell cycle progression plays a critical role in driving timely progression through G1/S and G2/M transitions in a kinase-dependent and -independent manner through interaction with CDK2/cyclinA and Pin1 [40]. In this regard, GRK2 levels are controlled normally by cell-cycle machinery and in response to DNA damage and differentially contribute either to cell cycle progression or cell arrest in a receptor-independent manner. When DNA is damaged, the pathways can be disrupted and in this case GRK2 can promote increased cell survival as a proarresting factor (see Figures 1 and 2). GRK2 protein levels are transiently downregulated during the G2/M transition through CDK2-mediated phosphorylation of GRK2, and preventing GRK2 phosphorylation impedes normal GRK2 downregulation and markedly delays cell cycle progression [40]. Of importance is GRK2 protein decay in G2, which is prevented in the presence of DNA damaging agents that trigger cell cycle arrest. Moreover, in cells with higher steady-state levels of the kinases, increased stabilized GRK2 levels inversely correlate with the p53 response and the induction of apoptosis [40]. Conversely, GRK2 is reported to cooperate with known oncogenes in transformation assays [44] and GRK2 has regulatory roles, which depend on extrinsic cues promoting cell division, as the GRK2-mediated phosphorylation of Hedgehog/Smoothened pathway triggers control of cell proliferation to promote Smo activity and relieve the Patched-dependent inhibition of cyclin B through Hedgehog ligand [45].

Certain signaling pathways instrumental in many cancers cause the upregulation of GRK2 protein levels in malignant cell lines [46, 47]. It is known that altered GRK2 expression levels modulate chemokines-mediated induction of MEK/ERK activity through both kinase-dependent and -independent function [48] and its aberrant epithelial cell motility that plays a key role in cancer progression and metastasis (Figure 1). GRK2 protein levels have been differentially upregulated in tissue samples of patients with granulose cell tumors, with differentiated thyroid carcinoma [49, 50] or downregulated in a subgroup of prostate tumors [51], which suggests that altered GRK2 expression in specific tumor cells may affect migration in response to particular stimuli and plays a critical role in basic cellular functions such as cell proliferation, differentiation or migration during development. Further, GRK2 inhibits TGF-mediated cell growth arrest and apoptosis in human hepatocarcinoma cells [46]. On the other hand, GRK2 attenuates serum- or PDGF-induced proliferation of thyroid cancer cell lines [49] and smooth muscle cells [43], whereas its expression increases MAPK signaling in response to EGF in HEK-293 cells [52] and GRK2 kinase activity is required for IGF-1-triggered proliferation and mitogenic signaling in osteoblasts [53] (Figures 1 and 2).

## 5. The Role of NOSs and ET in Liver Colorectal Metastatic Tumors

The absence of perivascular nerves in tumor vessels suggests that endothelial derived vasoactive substance NO and ET-1 may be the key factors in controlling tumor blood flow during tumor growth and metastasis [2]. In our earlier study, the ultrastructural distribution of different NOS isoforms and ET-1 immunoreactivity in human colorectal metastatic tumor liver was identified to know the role of NOSs and ET-1 in the pathophysiology of colorectal metastatic tumors by using preembedding peroxidase-anti-peroxidase (PAP) and postembedding immunoelectron microscopic triple gold labeling techniques [2].

**Figure 3:**
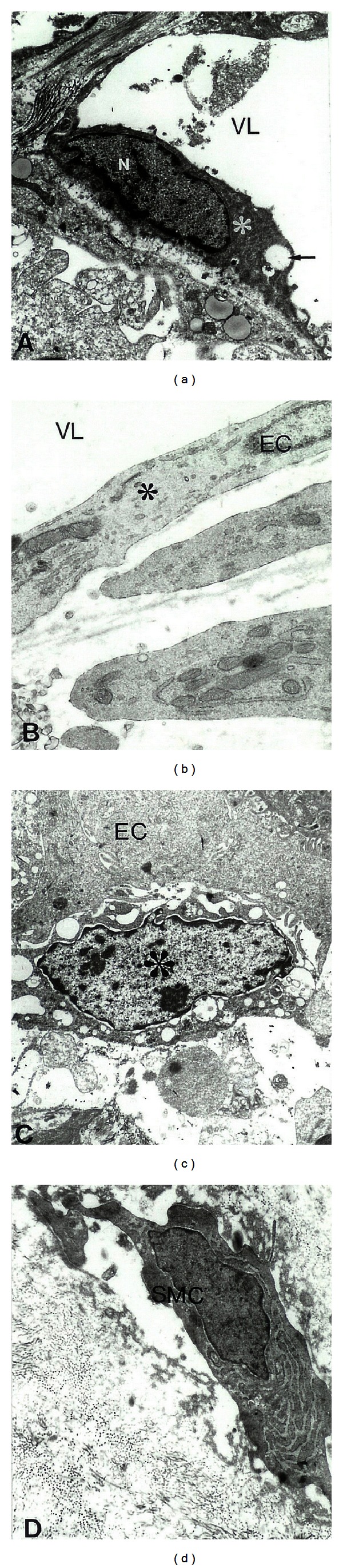
Electron Microscopic Peroxidase-anti-Peroxidase immunocytochemical determination of the distribution of NOS1 immunolabeling features in control (a) and metastatic colorectal cancer liver tumor tissues (b)–(d). (a) NOS1 immunopositive EC (indicated by asterisk) were seen in control liver microvessels. Vacuoles are indicated by single arrow X4 000. (b) Tumor vessel endothelium (indicated by asterisk) showed no staining for NOS1 antibody. X20,000. (c) NOS1 immunopositive white blood cells (indicated by asterisk) were attached to vessel endothelium in tumor growth regions. X6,000. (d) NOS1 immunopositive myofibroblast (smooth muscle cell) were seen in metastatic liver tumor tissues. X10,000. Reprinted with permission of J Submicrosc Cytol Pathol [2].

Electron Microscopic PAP techniques determination of the distribution of NOS1 immunolabeling features in control (Figure 3(a)) and metastatic colorectal cancer liver tumor tissues (Figures 3(b)–3(d) showed that NOS1 immunopositive EC were seen in control liver microvessels. In contrary to these observations, tumor vessel endothelium showed no staining for NOS1 antibody (Figure 3(b)). However, presence of the NOS1 immunopositive white blood cells was attached to vessel endothelium in tumor growth regions often observed (Figure 3(c)). In addition, NOS1 immunopositive myofibroblast (smooth muscle cell) was also seen in metastatic liver tumor tissues (Figure 3(d)).

**Figure 4:**
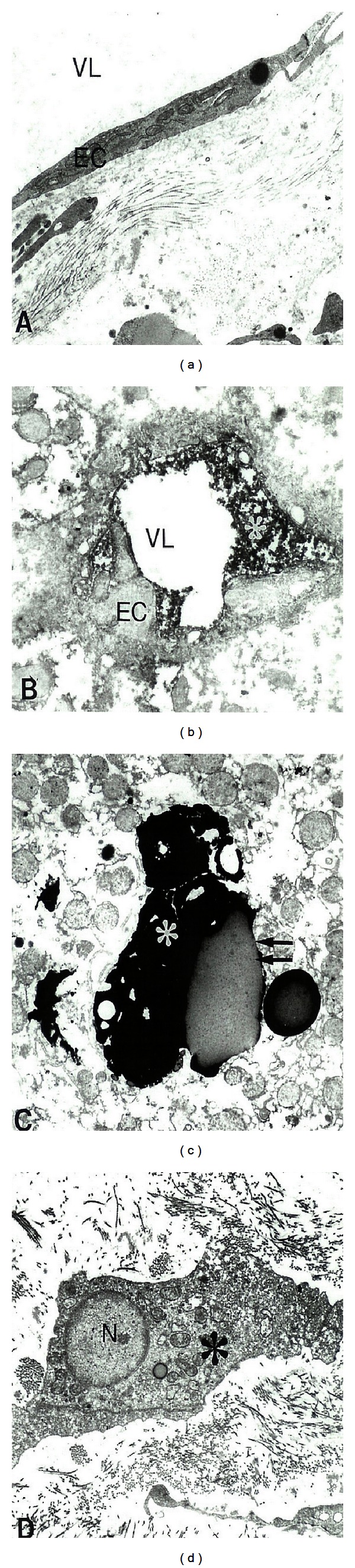
Ultrastructural labeling of inducible NOS (NOS2) immunoreactivity in metastatic liver tumor tissues that determined by using Pre-embedding Peroxidase-anti-Peroxidase Electron Microscopy Immunocytochemical techniques. (a) Tumor vessel EC was positively stained with NOS2. X8,000. (b) A high intensity of NOS2 immunopositive precipitate accumulated close to the luminal plasmalemma of the vascular EC in the tumor growth region. X8,000. (c) NOS2 immunopositive hepatocytes (asterisk). Lipid-contained areas of the hepatocytes (double arrow) were free from immunopositive reaction. X6,000. (d) NOS2 immunopositive myofibroblast-like cells (asterisk). X8,000. Reprinted with permission of J Submicrosc Cytol Pathol [2].

Ultrastructural labeling of inducible NOS (NOS2) immunoreactivity in metastatic liver tumor tissues determined by using electron microscopy and PAP immunocytochemical techniques showed that almost all of tumor vessel EC was positively stained with NOS2 (Figure 4(a)). Very often a high intensity of NOS2 immunopositive precipitate accumulated close to the luminal plasmalemma of the vascular EC in the tumor growth region (Figure 4(b)), indicating the elevated tissue levels of NO and ET-1 [2, 54]. The presence of NOS2 immunopositive hepatocytes and myofibroblast-like cells is also seen throughout the tumor growth area (Figure 4(c)). However, the lipid-contained areas of the cells were free from PAP immunopositive reaction (Figures 4(c)-4(d)).

**Figure 5:**
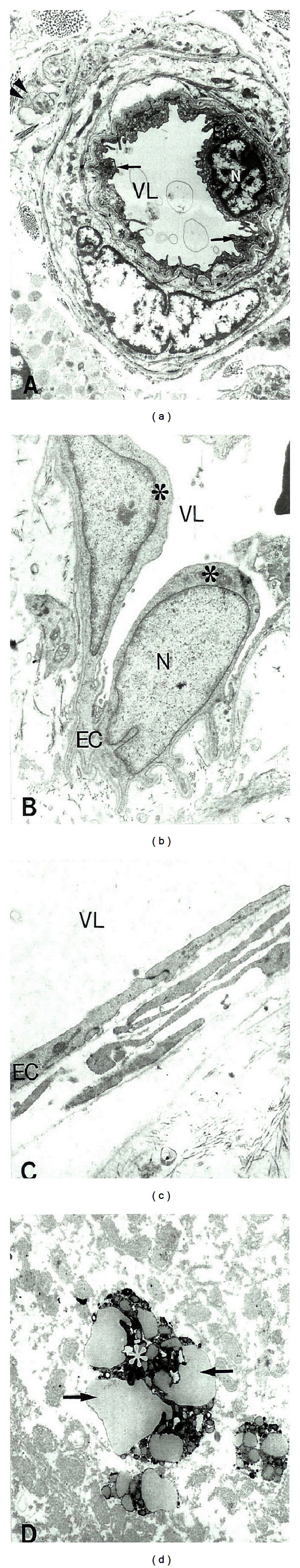
Ultrastructural features of endothelial specific NOS (eNOS or NOS3) labeling in control (a) and metastatic liver tumors tissues (b–d) determined by using Peroxidase-anti-Peroxidase Immunocytochemistry. (a) A large number of NOS3 immunopositive EC (indicated by arrows) were seen in control liver microvessels. X5,000. (b) NOS3 immunostaining was absent in EC (asterisk) in tumor vessels. X8,000. (c) EC from tumor microvessels did not show the presence of any NOS3 immunopositive reaction. X10,000. (d) NOS3 immunopositive hepatocytes (white asterisk) in metastatic liver tumors were seen. Lipid-enriched areas were free from NOS3 positive precipitate (arrows). X8,000. Reprinted with permission of J Submicrosc Cytol Pathol [2].

Ultrastructural features of endothelial specific NOS (eNOS or NOS3) labeling in control (Figure 5(a)) and metastatic liver tumors tissues (Figures 5(b)–5(d)) determined by using PAP method shows the presence a large number of NOS3 immunopositive EC in control liver microvessels. NOS3 immunostaining was absent in EC in tumor vessels (Figures 5(b)-5(c)). However, the presence of NOS3 immunopositive hepatocytes in metastatic liver tumors was seen. Lipid-enriched areas were free from NOS3 immunopositive precipitate (Figure 5(d)).

**Figure 6:**
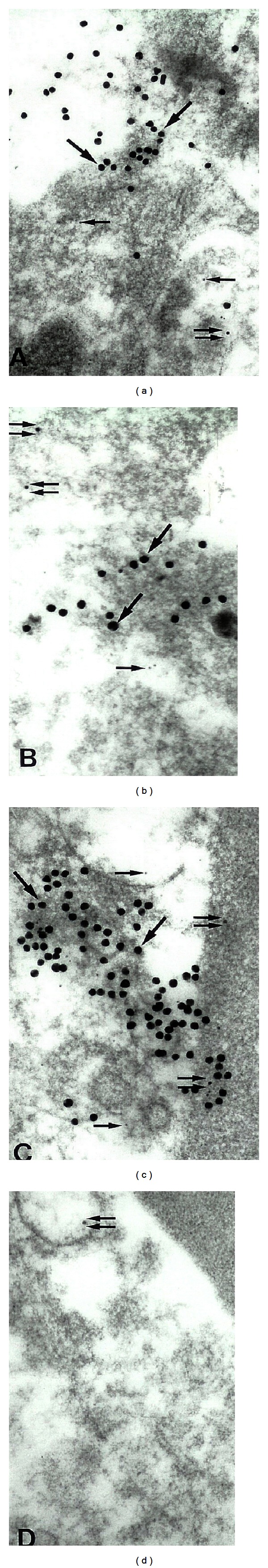
The distribution of NOS1-3 and ET-1 immunopositive gold particles in metastatic tumor vessel endothelium and hepatocytes that determined by using Post-embedding triple immunogold labeling techniques. (a) Clusters of NOS2 positive gold particles (20 nm, thick arrows) but not NOS3 (5** **nm, single thin arrow) and ET-1 (10** **nm, double arrow) were seen in tumor vessel endothelium. X100,000. (b) The expression of NOS1 (20 nm single arrow) was seen in the matrix of lipid laden hepatocytes in tumor growth area. NOS3 (10** **nm) and ET-1 (5** **nm) positive gold particles indicated by double and thin single arrow. X100,000. (c) Clusters of ET-1 (20 nm, single arrows) but not NOS1 (10 nm, double arrow) and NOS3 (5** **nm, single thin arrow) positive gold particles in the cytoplasmic matrix of hepatocytes were seen. X100,000. (d) EC from metastatic liver microvessels prepared as negative controls (through omission of the primary antibody) showed only the presence of single gold particles (10 nm, double arrow). X100,000. Reprinted with permission of J Submicrosc Cytol Pathol [2].

Our extended study by using postembedding triple immunogold labeling techniques showed that the clusters of NOS2 positive, but no NOS3 and ET-1 immunopositive containing gold particles were seen in tumor vessel endothelium (Figure 6(a)). The expression of NOS1 containing positive gold particles was seen in the matrix of lipid laden hepatocytes in tumor growth area (Figure 6(b)). Very often the clusters of ET-1 but not NOS1 and NOS3 positive gold particles in the cytoplasmic matrix of hepatocytes were seen (Figure 6(c)). EC from metastatic liver microvessels prepared as negative controls (through omission of the primary antibody) showed only the presence of single gold particles (Figure 6(d)). Our study highlights mitochondria as a critical constituent responsible for cell viability, which can be considered as a new research focus and of new diagnostic criteria for the earlier detection of tumors as well as treatment strategies at least in some tumors. However, further study needs to be carried out in order to clarify the exact nature of these relationships during the metastases and growth of primary and/or metastatic tumors.

## 6. Mitochondrial Lesions and Oncogenic Signaling Cascades

Aliev and coworkers' ongoing studies suggest that the mitochondrial lesions are the hallmarks of the primary glioblastoma (Aliev et al., unpublished observation). Vessel endothelium from tumor tissues shows the damage of mitochondrial cristae. The mitochondria-derived lysosomes appear to be a permanent feature of the glioma-derived tumor cells. Lipid laden tumor cells and surrounding cells often display varying degrees of mitochondrial abnormalities (such as mitochondria with broken cristae, presence of edema in their matrix, disruption of inner, and external mitochondrial membrane). Moreover, giant mitochondria also appear to be permanent features of tumor growth and metastases [2]. Comparatively characteristics of marginal and central portion of tumor tissues obtained from patients undergoing surgery with diagnosis of the primary glioblastoma shows that distance area of tumor tissue characterized heterogeneous distribution of damage in the structure of the mitochondria. Central regions of tumor tissues almost in all areas show astrocytes with clusters of mitochondria-derived lysosomes (Aliev et al., unpublished data). The same patterns of cellular and subcellular damage were also seen in spinal cord tumor (Aliev et al., unpublished data).

For the detection of mitochondrial DNA over-proliferation and deletion in tumor cells in AD tissues, Aliev and coworkers performed *in situ* hybridization [1, 32]. These studies demonstrated that successful dysregulation of cell cycle, and that early cell-cycle pathophysiology in AD may recruit oncogenic signal transduction mechanisms, which may be viewed as an abortive neoplastic transformation prominent during tumorigenesis and AD. These results also demonstrated that abnormal mitochondria and lipofuscin is a feature of hippocampal damaged neurons in human AD and aged AD transgenic (Tg+) mice that mimics human AD, and suggest a direct relationship between vascular abnormalities, BBB breakdown, neuronal loss, and amyloid depositions [1, 32]. The giant and electron dense mitochondria were reported to be a permanent feature of neuronal abnormality [1, 32]. *In situ* hybridization analysis with mouse and human mtDNA probes showed a large amount of mtDNA deletion in YAC-A*β*PP mice hippocampus compared with aged controls. The majority of these mtDNA deletions were found in mitochondrial-derived lysosomes in regions closely associated with lipofuscin, which suggests that proliferation, deletion, and duplication of mtDNA occurs in mitochondria, many of which have been fused with lysosomes in human AD [55–58], and transgenic mice as a model for neurodegeneration [1, 32, 57, 58]. Moreover, biopsy samples of AD patients were dominated by abnormal mitochondria as compared to control group. In one study, ultrastructural localization of mtDNA by *in situ* hybridization with colloidal gold showed that deleted mtDNA is mainly found in abnormal mitochondria [55]. The common features of the mitochondrial abnormality were seen in the brain during the tumorigenesis and in AD, indicating that most likely mitochondrial DNA overproliferation/deletion appeared to be key initiating factors for tumor growth/metastases [1, 32, 57, 58]. Therefore, investigating mitochondrial abnormality may open new windows not only for the better understanding of tumor pathogenesis but also for developing new treatment strategies.

Of particular importance, the effect of mitochondrial failure during tumor growth and metastases is dependent on the following factors: oxygen deficient tissue, NOSs enzymes activity, oxidative stress, cellular changes (hepatocytes, vascular, neuronal, and glial changes), and on the concomitant mitochondrial lesions and decline in normal organ function [1, 32, 57]. Upregulation of NOSs' enzyme activity induces formation of a large amount of reactive oxygen species (ROS). This may be a key factor in mitochondrial damage and energy failure occurring during carcinogenesis. Chronic hypoxia, a predominant characteristic of tumors, initiates the mitochondrial DNA overproliferation/deletion that then induces formation of large quantities of unwanted free radicals with concomitant energy deficiency (Aliev unpublished observations).

Some of the mitochondria mechanisms, which are incidentally heavily involved in the generation of ROS, result in oxidative damage to the vascular endothelium, as well as to other cellular constitutes in tumor tissues. Such changes also accompany tumor pathology. Previous studies demonstrated how age affects mitochondrial DNA mutations and overproliferation in liver and brain. Brain disorders that involve chronic hypoperfusion may be responsible for concomitant energy failure and the pathogenesis that underlies both disease processes, as hypoperfusion appears to induce oxidative stress, which is largely from ROS as well as NO [3].

However, these underlying processes also play a role not only in aging and age-associated diseases, but in tumor growth and metastases. Over the time, these processes initiate mitochondrial failure, a known factor in early AD pathogenesis [1, 32, 58]. In addition, NO can be produced for 80 years by neurons in human brain without any toxicity. Paradoxically, the production of the same molecule can become highly damaging to the same neurons within a few minutes during pathological challenges as occur after cerebral ischemia. The reaction of NO with superoxide (O_2_^−^) to form the much more powerful oxidant peroxynitrite (ONOO^−^) is a key element in resolving the contrasting roles of NO in physiology and pathology (vide supra). Future studies comparing the spectrum of mitochondrial damage and the relationship to NO-dependent oxidative stress-induced damage during the aging process [1, 32] and more importantly, during tumor development and metastasis are warranted [2].

Various studies demonstrated the involvement of NO in apoptosis and show that inhibition of mitochondrial respiration by NO results in a relative degree of mitochondrial hyperpolarization, an occurrence that requires the production of glycolytic ATP [6]. This observation indicates that the hyperpolarization may be a protective mechanism since neurons, and perhaps, other cells which do not utilize the glycolytic pathway and do not respond to NO by mitochondrial hyperpolarization, are more susceptible to NO-induced apoptosis than are glycolytically-active astrocytes (for a review, see [6]). Persistent inhibition of respiration by NO over a prolonged time eventually result in the collapse of membrane potential, ATP depletion and, ultimately, cell death (for a review, see [6]). NO may reversibly inhibit enzymes with transition metals or with free radical intermediates in their catalytic cycle. NO in micromolar concentrations reversibly inhibit catalase and cytochrome P-450 (for a review see [7]), which may transiently increase the leakage of superoxide from the electron transport chain. The superoxide so formed could then react with NO to generate peroxynitrite, which would cause irreversible injury to the mitochondria (for a review, see [7]). It can also inhibit ribonucleotide reductase, the enzyme responsible for DNA synthesis that contains a tyrosine radical. Large continuous fluxes of NO are necessary to inhibit ribonucleotide reductase, which would occur only under major inflammatory conditions or in the neighborhood of an activated macrophage. Indeed, activated macrophages produce both NO and superoxides, so the inactivation of mitochondria in tumor cells could well have been mediated by peroxynitrite (for a review, see [7]).

An increase in the release of NO from the vascular endothelium and other tumor tissue cells, including the natural killer cells (NKH-lymphocytes), can promote anti-tumor growth activity. High NO concentrations are generally tumoricidal as it inhibits DNA synthesis [59, 60]. NO, through reactions from the products of mitochondrial electron transport chain, produces ROS and RNS, which in sufficiently high concentrations cause DNA damage and apoptosis. Whereas DNA damage in cancer cells helps prevent cancer metastasis, it results in neuronal loss in AD.

Our report demonstrated that metastatic colorectal cancer to liver and malignant brain cancer are characterized by overexpression of several NOS enzymes, which coexist with mitochondrial ultrastructural alterations in tumor cells. Moreover, the degree of tumor growth and metastasis is linearly correlated with the overexpression of iNOS and increased level of ET-1 immunoreactivity [2]. The role of ET-1 as a mitogen in the pathogenesis of tumor growth and metastasis has been studied extensively [2, 59, 60]. Aliev and coworkers recorded expression of ET-1 immunoreactivity not only in vascular endothelium, but also in tumor cells, activated lymphocytes, SMC, and in liver hepatocytes [2, 54]. ET-1 has been reported as a mitogenic factor against a variety of cell types including the human hepatocellular carcinoma [61–64]. Studies by Nelson and coworkers [59, 60] found that circulating plasma ET-1 was elevated in more than half of men with advanced metastatic prostate cancer (PCA). The elevation of plasma ET-1 levels has been reported in hepatocellular carcinoma [64], but all patients in that study also had cirrhosis, which is independently associated with elevations in plasma ET-1 [65]. In many tissues, cellular overexpressions of ET-1 mRNA transcripts are in close proximity with those possessing ET-1 receptors [66, 67]. It has been suggested that the increased ET-1 immunoreactivity can be used as a marker for tumor growth and metastases [2, 59, 60]. However, the exact cellular mechanisms behind tumor vascular growth and the relation to NO oxidation products identified as nitrotyrosine formation, lipid peroxidation, ET-1 activity or mtDNA deletion remain to be unknown [2, 54]. Future studies comparing the spectrum of mitochondrial damage and the relationship to NO-dependent oxidative stress-induced damage during the aging process [1, 32, 57] and more importantly, during development and metastasis of tumor are in need of the hour. In addition, it has been also suggested that NO influences cellular differentiation through induction of gene expression [67]. This is interesting because a constitutively expressed NOS2 has been described in a colorectal adenocarcinoma cell line [68]. NO produced by stimulated macrophages [69] or released by NO donor drugs [70] inhibits tumor cell growth. Earlier, Aliev and coworkers have highlighted rise of NOS2 immunoreactivity as a hallmark in human metastatic colon cancer [2]. However exact role of NOS2 activity on the mitochondrial lesions and/or mitochondrial DNA overproliferation and/or deletion in these conditions are unknown. The increased understanding of the relationship between the degree of mitochondrial lesions, NOS and nitrotyrosine protein overexpression, and mitochondrial DNA overproliferation/deletion, could give us a better understanding of tumor pathogenesis. This may eventually lead to new and effective treatments strategies. For example, if the degree of pathology can be correlated with the quantity of the NOS enzymes, immunoreactivities expressed and mtDNA overproliferation/deletion, then manipulating the systems metabolically may be sought which can lead to early death of the injured cancer cell mitochondria. Moreover, mitochondria appear to be primary targets for apoptotic cell death. Moreover, involvement of NO has already been demonstrated in apoptosis and it has been shown that inhibition of mitochondrial respiration by NO results in a relative degree of mitochondrial hyperpolarization, an occurrence that requires the production of glycolytic ATP [6].

Cytokines increase NOS2 mRNA levels in macrophages, hepatocytes, and vascular SMC's in a dose- and time-dependent manner [71]. An alternative explanation for the increased NOS2 expression in vascular endothelium and all NOS isoforms immunoreactivity in other cells in metastatic liver tissue is that a large number of noncontractile types of fibroblasts and/or myofibroblast-like cells are present in tumor tissues. The marginal increase in NOS2 immunoreactivity in tumor vessel endothelium, along with the increased expression of all three NOS isoforms in other cells in liver, were associated with a significant overexpression of ET-1 immunoreactivity in all tumor tissue cells. Our report indicated that NOS2 and ET-1 expressions are linearly correlated with the degree and nature of tumor growth [2, 54]. It is interesting that the total number of NOSs (NOS 1–3) immunopositive EC is nearly equal to the total number of ET-1 immunopositive EC. We speculate that most likely this positive feedback appears to be a compensatory action of tumor invaded organs during the tumor growth and metastases.

## 7. Oncogenic Parallelism between Cancer and AD: Potential Common Treatment Strategies

When cells receive growth stimuli, or mitotic drive, they upregulate cyclin-dependent kinases (CDKs) and their cognate activating cyclins to orchestrate DNA replication, cytoskeletal reorganization and cellular metabolism required for proliferation (Table 1). Hormonal signals from luteinizing hormone and other hormones can contribute to this mitotic drive [72]. Mitotic drive and the orderly progression through cell cycle, involve cyclins and CDKs which form complexes that are able to phosphoregulate a wide variety of substrates [73]. However, extrinsic mitotic pressures and proper cell cycle progression can also involve resensitization. Association with AD and AD-related cytoskeletal pathology [74] may be involved in aberrant neuronal sprouting response [75–78]. Mitotic drive may arise from inflammatory processes, oxidative stress and other excitatory stressors [79–81]. Strong support exists in literature for an AD-cell cycle-associated emergence from a quiescent state and researchers have looked at it as a recapitulation or vestige of an evolutionarily conserved process [82, 83] (Table 1). Recently reversion in AD pathology has been demonstrated by anticancer drugs [84]. Furthermore, AD-associated proteins and the cell cycle activation from mitotic drive are intimately linked to tau proteins as well as to A*β*, the extracellular lesion associated with the disease [85–90]. NO has both genotoxic and angiogenic properties and has been reported to inhibit the release of mitogen from platelets. Another strategy for tumor treatment has focused on the inhibition of tumor angiogenesis. It has been well established that angiogenesis is a critical event in tumor growth and metastasis [91]. Increased NO production may selectively support mutant p53 cells and may also contribute to tumor angiogenesis by upregulation of vascular endothelial growth factor [92]. There is a growing scientific agreement that antioxidants, particularly the polyphenolic forms, may help lower the incidence of disease, such as certain cancers, cardiovascular, and neurodegenerative diseases, DNA damage, or even have antiaging properties. On the other hand, questions remain as to whether some antioxidants or phytochemicals potentially could do more harm than good, as an increase in glycation-mediated protein damage (carbonyl stress) and some risk has been reported. A recent review highlights both anti- and prooxidant properties associated with polyphenolic compounds [93]. Nevertheless, the quest for healthy aging has led to the use of antioxidants as a means to disrupt age-associated deterioration in physiological function, dysregulated metabolic processes, or prevention of many age-related diseases. Although a diet rich in polyphenolic forms of antioxidants does seem to offer hope in delaying the onset of age-related disorders, it is still too early to define their exact clinical benefit for treating age-related diseases. Regardless of where the debate will end, it is clear that any deficiency in antioxidant vitamins or adequate enzymatic antioxidant defenses can shift the redox balance in some diseases [94].

## 8. Conclusion and Future Remarks

The absence of neuronal control (e.g., perivascular nerves) in tumor vessels suggests that endothelial-derived vasoactive substance, namely NO and ET-1, may be key factors in controlling tumor blood flow during tumor growth and metastasis. An imbalance between endothelial-derived vasoconstrictors and vasodilators, along with deficiency of antioxidant systems may result in mitochondria lesions in tumors. NO-induced mitochondrial failure is a causative factor in the pathogenesis of tumors, especially tumor angiogenesis. Conversely, recent studies have shown that apart from vasodilator and antiplatelet activities, there are other actions of NO that might be regarded as “*antiatherogenic*” (probably also “*antiangiogenic*”). NO has both genotoxic and angiogenic properties and has been reported to inhibit the release of mitogen from platelets. Another strategy for tumor treatment has focused on the inhibition of tumor angiogenesis. Increased NO production may also contribute to tumor angiogenesis by upregulation of vascular endothelial growth factor. We hypothesize that mitochondrial involvement in this cascade may be a major factor that controls tumor growth and metastasis. Future studies of mitochondrial pathophysiology in various benign and malignant tumors, including colorectal, liver, and brain cancer may provide new insights in carcinogenesis and may lead to rational targets and strategies for better and more effective cancer treatments.

## Figures and Tables

**Table 1:** 

Marker	Role	Association with Alzheimer disease
Cyclin A	S to G2/M	[95, 96]
Cyclin B	G2/M	[97–99]
Cyclin C	No known role	
Cyclin D (D1, D3)	G0/G1/lateG1/S	[98–101]
Cyclin E	G1 to G1/S	[97, 102]
p34cdc2/cdk 1	Late G2/M	[95, 96, 103, 104]
Cdk4/Cdk6	G1/G1/S	[105–108]
Cdk5/p25/p35	G2 D1, D3 G1 Cyclins	[109–114]
Nclk cdc2-like kinase	Cyclin A kinase	[109, 115, 116]
Cdk7/MPM2	CDK activated kinase	[101, 117]
Cdc42/rac	GTPase/cell division	
p21ras	G protein/MAPK	[118, 119]
MRG 15	M phase regulator	[120]
Ki-67	LateG1,S,G2,M	[97, 120]
p105/pRb	G2/M TF	[105, 120]
PCNA	non cell-cycle specific	[99]
p107/pRb	Cdk2/4/6, check pt	[77, 105] (negative association)
c-myc	S to G2 checkpoint	[77] (negative association)
p53/MDM2	Repressor complex	[118, 120, 121]
ATM	Check-point	[77]
Raf/Raf-1	Check point kinase	[117]
p16INK4a p18p15p19	CyclinD/cdk4/6 inhibitors of M phase	[101, 122]
p27/Kip1	Cyclin D and E/cdk7 inhibitor	[77] (negative association), [101]
WAF-1/p21/Cip1	Multi-Cyclin/cdk-inhibitor (G1 and S)	[118]
Plk1/cdc5 Polo-like kinase	G2/M M check point	[123]
PP2A or PP2B	Phosphatase (Cdk5, cdc2)	[124–126]
PP-1		[112, 124]
Cdc25 Cdc25A	Phosphatase G2/M	[127, 128]
PKC/Wnt path	Translation control	[129–132]
PKA	Kinase	[133, 134]
PKN	Kinase	[135]
PI3K	Kinase	[136, 137]
AKT/PKB/RAC	Kinase	[135, 138, 139]
TGFBeta/TAK	Kinase	[140, 141]
p44/p42 MAPK (ERK1/2)	MAP kinase	[95, 142–146]
CamK	Kinase Ca/calmodulin regulated	[147]
p38 MAPK	Kinase	[146, 148, 149]
JNK/(SAPK-2/3)-alpha gamma	Kinase (stress activated)	[146, 150]
MEK	MAPK kinase	[122, 151]
GSK-3 and beta Catenin	Proline-dependent protein kinase (PDPK)	[111, 112, 131, 138, 152–157]
P120/E-cadherin	Adhesion complex	[158]
c-fos	TF/regulator	[159]
14-3-3/14-3-3zeta	Adaptor protein	[160, 161]
c-jun/p38, AP-1	TF component	[159, 162, 163]
Fyn	Transcription factor	[164, 165]
p53	TF/DNA damage	[97, 166]
Rho	G-protein	[135]
Rap Rab	G-protein	[117]
Sos-1	Guanine nucleotide exchange factor	[167]
Grb-2	Adaptor	[167]

Modified and reprinted with permission of CNS Neurol Disord Drug Targets [92]
